# Depression in non-alcoholic fatty liver disease is associated with an increased risk of complications and mortality

**DOI:** 10.3389/fmed.2022.985803

**Published:** 2022-10-06

**Authors:** Cheng Han Ng, Jieling Xiao, Nicholas W. S. Chew, Yip Han Chin, Kai En Chan, Jingxuan Quek, Wen Hui Lim, Darren Jun Hao Tan, Ryan Wai Keong Loke, Caitlyn Tan, Ansel Shao Pin Tang, Xin Lei Goh, Benjamin Nah, Nicholas Syn, Dan Yock Young, Nobuharu Tamaki, Daniel Q. Huang, Mohammad Shadab Siddiqui, Mazen Noureddin, Arun Sanyal, Mark Muthiah

**Affiliations:** ^1^Yong Loo Lin School of Medicine, National University of Singapore, Singapore, Singapore; ^2^Department of Cardiology, National University Heart Centre, National University Hospital, Singapore, Singapore; ^3^Division of Gastroenterology and Hepatology, Department of Medicine, National University Hospital, Singapore, Singapore; ^4^National University Centre for Organ Transplantation, National University Health System, Singapore, Singapore; ^5^Department of Gastroenterology and Hepatology, Musashino Red Cross Hospital, Tokyo, Japan; ^6^Division of Gastroenterology, Hepatology and Nutrition, Department of Internal Medicine, Virginia Commonwealth University, Richmond, VA, United States; ^7^Houston Research Institute, Houston, TX, United States

**Keywords:** depression, NAFLD, NASH, NHANES, complication, mortality

## Abstract

**Background and aims:**

The global prevalence of non-alcoholic fatty liver disease (NAFLD) is expected to rise continuously. Furthermore, emerging evidence has also shown the potential for concomitant depression in NAFLD. This study aims to examine the prevalence, risk factors, and adverse events of depression in NAFLD and evaluate whether treated depression can reverse the increased risks of adverse outcomes.

**Materials and methods:**

This study analyses the 2000–2018 cycles of NHANES that examined liver steatosis with fatty liver index (FLI). The relationship between NAFLD and depression was assessed with a generalized linear mix model and a sensitivity analysis was conducted in the no depression, treated depression, and untreated depression groups. Survival analysis was conducted with cox regression and fine gray sub-distribution model.

**Results:**

A total of 21,414 patients were included and 6,726 were diagnosed with NAFLD. The risk of depression in NAFLD was 12% higher compared to non-NAFLD individuals (RR: 1.12, CI: 1.00–1.26, *p* = 0.04). NAFLD individuals with depression were more likely to be older, females, Hispanics or Caucasians, diabetic, and have higher BMI. Individuals with depression have high risk for cardiovascular diseases (CVD) (RR: 1.40, CI: 1.25–1.58, *p* < 0.01), stroke (RR: 1.71, CI: 1.27–2.23, *p* < 0.01), all-cause mortality (HR: 1.50, CI: 1.25–1.81, *p* < 0.01), and cancer-related mortality (SHR: 1.43, CI: 1.14–1.80, *p* = 0.002) compared to NAFLD individuals without depression. The risk of CVD, stroke, all-cause mortality, and cancer-related mortality in NAFLD individuals with treated depression and depression with untreated treatment was higher compared to individuals without depression.

**Conclusion:**

This study shows that concomitant depression in NAFLD patients can increase the risk of adverse outcomes. Early screening of depression in high-risk individuals should be encouraged to improve the wellbeing of NAFLD patients.

## Introduction

Non-alcoholic fatty liver disease (NAFLD) is the fastest growing cause of chronic liver disease affecting 25–33% of the prevalence and is expected to rise in conjunction with the current obesity pandemic (1–3). NAFLD comprises two subtypes including non-alcoholic fatty liver (NAFL) and non-alcoholic steatohepatitis (NASH) (4) with the latter associated with more adverse outcomes (5). However, there are currently no Food and Drug Administration (FDA) approved medications for NASH and liver transplant (6) is reserved for patients with end-stage liver disease (7, 8).

While the presence of NAFLD has been known to be associated with a host of extrahepatic complications including increasing the risk of cardiovascular disease (9), chronic kidney disease, osteoporosis (4) and malignancies, emerging evidence has since shown the potential for NAFLD to develop depression (10, 11). A recent meta-analysis by Xiao et al. involving 2,041,752 patients found that the presence of NAFLD was associated with a 1.29 increase in the odds of depression (OR: 1.29, CI: 1.02–1.64, *p* = 0.03). Additionally, a longitudinal study by Labenz et al. found a 21% increase in the risk of antidepressants use in NAFLD patients. The presence of depression in the general population has been associated with a plethora of health complications such as increased tendency for development of cardiovascular diseases (CVD), metabolic syndrome and heightened risk of mortality (12–15).

Yet, the impact of depression in NAFLD has not been well-examined with only a previous analysis by Sayiner et al. showing that depression in NAFLD increases the rate of all-cause mortality (16). Here, we sought to comprehensively examine the prevalence, risk factors, and related adverse events that depression poses to NAFLD individuals in the United States with data from the National Health and Nutrition Examination Survey (1999–2018) study. We additionally sought to examine the risks of all-cause mortality, competing for risk of cardiovascular mortality, and cancer-related mortality in NAFLD individuals with depression. Lastly, we examined treating depression in NAFLD can mitigate the increased risks of adverse outcomes.

## Materials and methods

### Study population

This study analyses the 2000–2018 cycles of NHANES carried out by the United States National Centre for Health Statistics by the Centre for Disease Control and Prevention (CDC). The NHANES study was a cross-sectional survey platform that adopted a stratified, multistage, clustered probability sampling design. Individual representatives of the general non-institutionalized population were identified and studied. It includes a structured interview conducted in the home and a standardized health examination conducted at a mobile examination center subsequently. The standardized health examination encompasses a physical examination and laboratory tests. The complete methodology of NHANES data collection was published previously (17). National Centre for Health Statistics Research Ethics Review Board has approved the original survey. As the data used in the analysis is de-identified and publicly available, review by the Institutional Review Board was not required. The study population for this study’s analysis included adult NAFLD patients, aged 18 years and older with fatty liver. Participants diagnosed with other etiologies of liver disease (alcohol, autoimmune, hepatitis B or C), retroviral disease, or participants without data on measurements of depression were excluded from the analysis. Patients with a positive human immunodeficiency virus diagnosis were also excluded from the analysis.

### Definitions

The definition of NAFLD was adapted based on the American Association for the Study of Liver Disease (AASLD) guidelines for NAFLD (18) and was defined as the presence of hepatic steatosis is the absence of substantial alcohol use (≥2 drinks a day in men, ≥3 drinks a day in women). The presence of steatosis was detected by Fatty Liver Index (FLI) and the United States Fatty Liver Index (US-FLI) where possible with a cut-off of 60 (19) and 30 (20), respectively. Overweight patients were defined as a BMI ≥ 25 for Caucasians and BMI ≥ 23 for Asians where data was available (21). Hypertension was defined as having a blood pressure of ≥140 mm Hg. Diabetes was defined as the presence of self-report presence of diabetes, glycohemoglobin ≥6.5%, or fasting plasma glucose ≥7 mmol/l. A diagnosis of depression was defined as the use of antidepressants or elevated depression scores on the Patient Health Questionnaire-9 (PHQ-9). The scoring system ranged from 0 to 27 with clinically significant depressive symptoms defined as scores ≥10 (22). The PHQ-9 adopted the modified criteria from the Diagnostic and Statistical Manual of Mental Disorders, 4th edition (DSM-IV), and used categorical algorithms to diagnose psychiatric disorders (23), and questions were asked at the Mobile Examination Centre by trained interviewers in face-to-face interview (24). A PHQ-9 score of ≥10 has a sensitivity and specificity of 88% for a clinical diagnosis of depression and has been a well-validated tool for identifying major depressive disorders (22, 25, 26). The subjects responded to the frequency in which they experienced depressive symptoms over the last 2 weeks, on a scale of 0–3. These symptoms included anhedonia, sleep disturbance, fatigue, depressed mood, affected appetite, low self-esteem, suicidal ideation, concentration challenges, and suicidal ideation. Adverse outcomes were defined as events including cardiovascular disease, cancer, stroke, chronic kidney disease, and mortality. The presence of cardiovascular disease, cancer, and stroke was retrospectively reported by patients based on a formal diagnosis from a physician. The presence of chronic kidney disease was defined as an estimated glomerular filtration rate of 60 and less calculated based on the Modification of Diet in Renal Disease (MDRD) formula. Mortality was identified from supplementary data in the national death index based on ICD-9 Codes.

### Statistical analysis

Analytical methods were designed with reference to previously published studies (27–29). Statistical analysis was conducted using STATA (16.1 StataCorp, Texas, United States). Descriptive statistics were summarized in median and interquartile range (IQR) for continuous variables and proportions with a 95% confidence interval for binary variables. A non-parametric Wilcoxon rank sum test and chi square test were used to compare continuous variables and dichotomous variables, respectively. A generalized linear mix model with a log link, gaussian family, and robust variance estimator was used to estimate the effect size of binary events in risk ratios (RR). A risk ratio is a more robust measure of binary events when the events are uncommon as opposed to an odds ratio. Mortality outcomes were examined with a cox proportional model for hazard ratios (HR) and violations of proportionality were examined with Schoenfeld residuals and a log-log plot. A separate competing risk analysis was used to examine the risk of CVD mortality and cancer-related mortality with the fine gray sub-distribution hazard ratios (SHR). A cluster analysis was also included in both RR, HR, and SHR based on the year of study to account for heterogeneity introduced by the year of study. Subsequently, a sensitivity analysis was conducted of the included population into 3 groups: (1) no depression, (2) treated depression, and (3) untreated depression. We defined treated depression as a PHQ-9 of <10 with antidepressants and untreated depression as a PHQ-9 ≥ 10 without treatment.

## Results

### Baseline characteristics of included population

**FIGURE 1:**
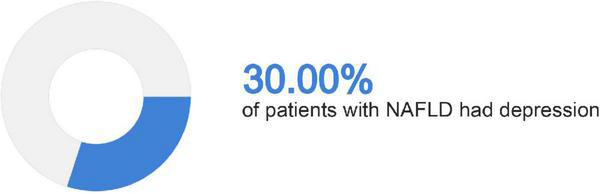
Proportion of NAFLD patients with depression.

A total of 21,414 patients were included in the analysis and 6,726 were identified to have NAFLD. The summary of baseline characteristics can be found in Table 1. NAFLD individuals were significantly older with diabetes and a higher BMI measurement. The use of antidepressants was similarly more common in NAFLD compared to non-NAFLD individuals. Of the 6,726 individuals with NAFLD, 2,017 (30%, CI: 28.9 to 31.1%) individuals had either an elevated PHQ-9 score suggestive of depression or reported use of antidepressants (Figure 1). After adjusting for cofounders in a generalized linear model with variables including age, gender, race, diabetes, overweight, and a cluster variable on the year of study, the risk of depression in NAFLD was 12% higher compared to non-NAFLD individuals (RR: 1.12, CI: 1.00 to 1.26, *p* = 0.04).

**TABLE 1:** Summary of baseline characteristics of included population.

	NAFLD	Non-NAFLD	*P*-value
Age (Years)	52.16 (IQR: 39.00 to 65.00)	44.81 (IQR: 28.00 to 60.00)	<0.01*
Diabetes (%)	0.29 (95% CI: 0.28 to 0.30)	0.12 (05% CI: 0.11 to 0.12)	<0.01*
HTN (%)	0.63 (95% CI: 0.62 to 0.64)	0.41 (95% CI: 0.40 to 0.41)	<0.01*
Body mass index (kg/m^2^)	34.68 (IQR: 30.17 to 37.80)	26.92 (IQR: 22.91 to 29.52)	<0.01*
Waist circumference (cm)	112.50 (IQR: 103.20 to 119.60)	93.60 (IQR: 82.90 to 101.50)	<0.01*
Weight (kg)	95.81 (IQR: 81.45 to 106.20)	75.53 (IQR: 62.15 to 84.90)	<0.01*
Platelet count	258.36 (IQR: 211.00 to 297.00)	250.11 (IQR: 206.00 to 287.00)	<0.01*
Glycohemoglobin	6.06 (IQR: 5.40 to 6.20)	5.58 (IQR: 5.10 to 5.70)	<0.01*
Fasting glucose (mmol/L)	6.69 (IQR: 5.44 to 6.77)	5.79 (IQR: 5.05 to 5.88)	<0.01*
Total bilirubin (μmol/L)	10.09 (IQR: 6.84 to 11.97)	11.40 (IQR: 8.55 to 13.68)	<0.01*
Total cholesterol (mg/dL)	198.21 (IQR: 169.00 to 224.00)	190.00 (IQR: 161.00 to 215.00)	<0.01*
LDL-Cholesterol (mg/dL)	116.37 (IQR: 91.00 to 138.00)	111.00 (IQR: 86.00 to 133.00)	<0.01*
Direct HDL-Cholesterol (mg/dL)	48.01 (IQR: 39.00 to 55.00)	55.52 (IQR: 44.00 to 65.00)	<0.01*
Triglycerides (mg/dL)	194.43 (IQR: 112.00 to 235.00)	133.75 (IQR: 71.00 to 158.00)	<0.01*
Depression score	5.29 (IQR: 2.00 to 7.00)	4.84 (IQR: 2.00 to 6.00)	<0.01*
**Antidepressants**			
Antidepressants Use (%)	0.20 (95% CI: 0.19 to 0.21)	0.15 (95% CI: 0.15 to 0.16)	<0.01*
No antidepressants use (%)	0.80 (95% CI: 0.79 to 0.81)	0.85 (95% CI: 0.84 to 0.85)	<0.01*
**Gender**			
Male (%)	0.39 (95% CI: 0.38 to 0.40)	0.46 (95% CI: 0.45 to 0.47)	<0.01*
Female (%)	0.61 (95% CI: 0.60 to 0.62)	0.54 (95% CI: 0.53 to 0.55)	
**Ethnicity**			
Mexican American (%)	0.17 (95% CI: 0.16 to 0.18)	0.15 (95% CI: 0.15 to 0.16)	<0.01*
Other Hispanic (%)	0.09 (95% CI: 0.09 to 0.10)	0.10 (95% CI: 0.09 to 0.10)	
Caucasians (%)	0.44 (95% CI: 0.43 to 0.46)	0.46 (95% CI: 0.45 to 0.46)	
African American (%)	0.21 (95% CI: 0.20 to 0.22)	0.19 (95% CI: 0.18 to 0.19)	
Other race (%)	0.08 (95% CI: 0.08 to 0.09)	0.11 (95% CI: 0.10 to 0.11)	

NAFLD, non-alcoholic fatty liver disease; LDL, low-density lipoprotein; HDL, high-density lipoprotein; HTN, hypertension; IQR, interquartile range; 95% CI, 95% confidence interval.

*Bolded *p*-value ≤ 0.05 denotes statistical significance.

### Factors associated with depression

Non-alcoholic fatty liver disease individuals with depression were more likely to be older, females, diabetics, and higher BMI (Table 2). Ethnicities were also influencing factors in the diagnosis of depression in NAFLD. In multivariate analysis, an older age (RR: 1.01, CI: 1.00 to 1.02, *p* < 0.01), female gender (RR: 1.42, CI: 1.32 to 1.52, *p* < 0.01), and diabetes (RR: 1.10, CI: 1.32 to 1.52, *p* = 0.03) increase the risk of depression in NAFLD. A higher BMI resulted in a marginal increase in the risk of depression (RR: 1.01, CI: 1.00 to 1.02, *p* < 0.01). With Mexican Americans as a reference, other Hispanics (RR: 1.17, CI: 0.98 to 1.39, *p* < 0.01) and Caucasians (RR: 1.49, CI: 1.35 to 1.63, *p* < 0.01) resulted in an increased risk of depression amongst individuals with NAFLD. Higher total cholesterol and triglyceride levels similarly result in a marginal increase in the risk of depression in NAFLD (RR: 1.01, CI: 1.00 to 1.02, *p* < 0.01) in multivariate analysis. Multivariate adjustments for hypertension, however, resulted in a significantly higher risk of depression in NAFLD (RR: 1.21, CI: 1.12 to 1.30, *p* < 0.01).

**TABLE 2:** Baseline characteristics of population with and without depression in NAFLD.

	Depression	No depression	*P*-value
Age (Years)	54.22 (IQR: 44.00 to 65.00)	51.27 (IQR: 37.00 to 65.00)	<0.01*
Diabetes (%)	0.32 (95% CI: 0.30 to 0.34)	0.27 (95% CI: 0.26 to 0.28)	<0.01*
HTN (%)	0.69 (95% CI: 0.67 to 0.71)	0.61 (95% CI: 0.59 to 0.62)	<0.01*
Body mass index (kg/m^2^)	35.15 (IQR: 30.50 to 38.40)	34.48 (30.00 to 37.51)	<0.01*
Waist circumference (cm)	113.36 (IQR: 104.00 to 120.60)	112.13 (IQR: 103.00 to 119.00)	<0.01*
Weight (kg)	95.68 (IQR: 81.10 to 106.60)	95.87 (IQR: 81.50 to 106.10)	0.88
Platelet count	263.97 (IQR: 213.00 to 302.00)	255.96 (IQR: 209.00 to 295.00)	<0.01*
Glycohemoglobin	6.10 (IQR: 5.40 to 6.30)	6.04 (IQR: 5.40 to 6.20)	0.17
Fasting glucose (mmol/L)	6.82 (IQR: 5.44 to 6.96)	6.63 (IQR: 5.44 to 6.72)	0.41
Total bilirubin (μmol/L)	9.75 (IQR: 6.84 to 11.97)	10.23 (IQR: 6.84 to 11.97)	<0.01*
Total cholesterol (mg/dL)	201.55 (IQR: 171.00 to 228.00)	196.78 (IQR: 167.00 to 222.00)	<0.01*
LDL-Cholesterol (mg/dL)	116.94 (IQR: 90.00 to 139.00)	116.11 (IQR: 91.00 to 138.00)	0.48
Direct HDL-Cholesterol (mg/dL)	48.74 (IQR: 40.00 to 57.00)	47.75 (IQR: 39.00 to 55.00)	<0.01*
Triglycerides (mg/dL)	200.03 (IQR: 119.00 to 241.00)	192.03 (IQR: 109.00 to 232.00)	<0.01*
Depression score	10.85 (IQR: 6.00 to 15.00)	3.40 (IQR: 2.00 to 5.00)	<0.01*
**Gender**			
Male (%)	0.29 (95% CI: 0.27 to 0.31)	0.43 (95% CI: 0.42 to 0.45)	<0.01*
Female (%)	0.71 (95% CI: 0.69 to 0.73)	0.57 (95% CI: 0.55 to 0.58)	
**Ethnicity**			
Mexican American (%)	0.13 (95% CI: 0.11 to 0.14)	0.18 (95% CI: 0.17 to 0.20)	<0.01*
Other Hispanic (%)	0.09 (95% CI: 0.08 to 0.11)	0.09 (95% CI: 0.09 to 0.10)	
Caucasians (%)	0.54 (95% CI: 0.52 to 0.56)	0.40 (95% CI: 0.39 to 0.42)	
African American (%)	0.18 (95% CI: 0.16 to 0.19)	0.23 (95% CI: 0.22 to 0.24)	
Other race (%)	0.06 (95% CI: 0.05 to 0.07)	0.09 (0.08 to 0.10)	

LDL, low-density lipoprotein; HDL, high-density lipoprotein; HTN, hypertension; IQR, interquartile range; 95% CI, 95% confidence interval. *Bolded *p*-value ≤ 0.05 denotes statistical significance.

### Complications in non-alcoholic fatty liver disease

#### Overall analysis of depression impact in non-alcoholic fatty liver disease

**FIGURE 2:**
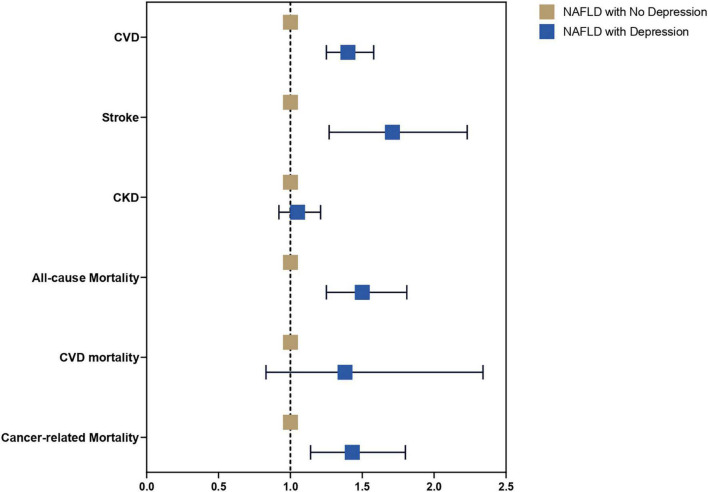
Forest plots of adverse outcomes in NAFLD with and without depression.

**FIGURE 3:**
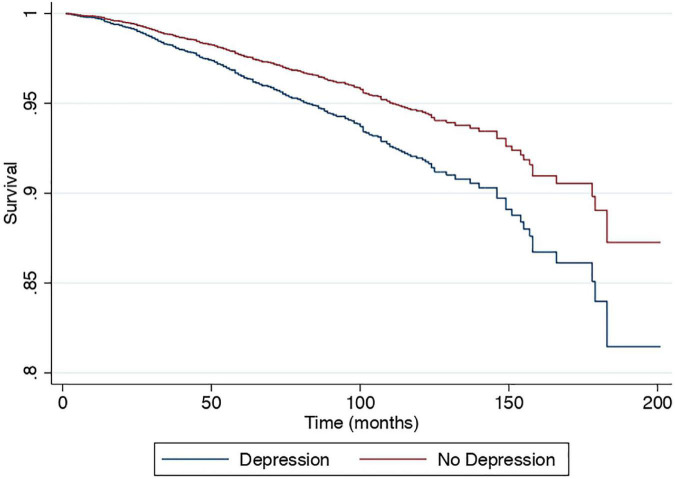
Cox proportional survival of all-cause mortality with and without depression.

A multivariate generalized linear model with a robust variance estimator adjusting for age, gender, race, BMI, and diabetes was used to compare the outcomes between individuals with and without depression in NAFLD (Figure 2). Individuals with depression were more likely to be associated with CVD (RR: 1.40, CI: 1.25 to 1.58, *p* < 0.01) and stroke (RR: 1.71, CI: 1.27 to 2.23, *p* < 0.01) but not CKD (RR: 1.05, CI: 0.92 to 1.21, *p* = 0.46) compared to NAFLD individuals without depression. A Cox proportional model was used to examine the risk of all-cause mortality, and there were no violations of the cox proportional model examined with Schoenfeld residuals and log-log plot (Figure 3). Individuals with depression were at a 50% increased risk of mortality (HR: 1.50, CI: 1.25 to 1.81, *p* < 0.01). There was no statistically significant increased risk of CVD mortality (SHR: 1.38, CI: 0.83 to 2.34, *p* < 0.01) compared to NAFLD individuals without depression. Cancer-related mortality, however, was higher in NAFLD individuals with depression (SHR: 1.43, CI: 1.14 to 1.80, *p* = 0.002).

#### Sensitivity analysis by treated and untreated depression

**FIGURE 4:**
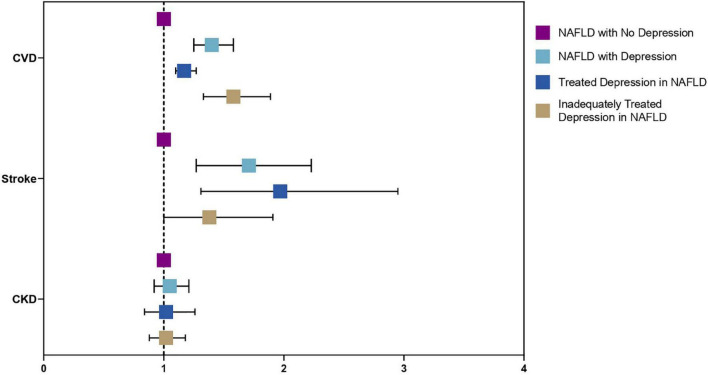
Forest plots of adverse outcomes in NAFLD with depression, without depression, treated, and untreated depression.

**FIGURE 5:**
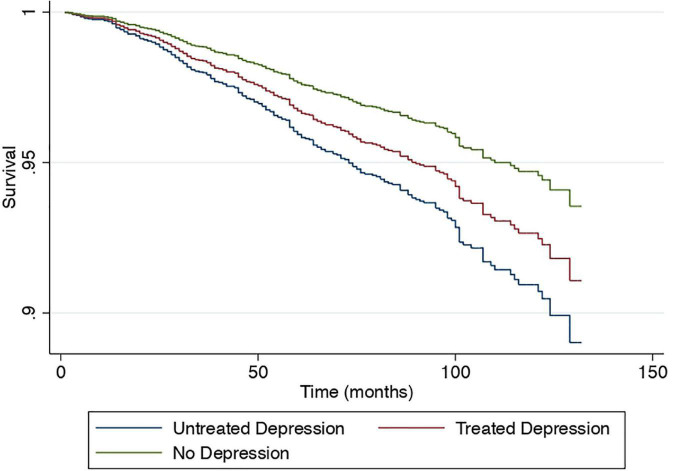
Cox proportional survival of all-cause mortality without depression, treated and untreated depression.

A sensitivity analysis was conducted to examine the effect of depression in a subdivided population of NAFLD including individuals without depression as a reference, treated depression, and untreated depression in NAFLD. Results from the multivariate generalized linear model with a robust variance estimator adjusted for age, gender, race, BMI and diabetes found a reduced magnitude of events with treated depression in NAFLD compared to untreated depression (Figure 4). The risk of CVD events in individuals with treated depression and untreated depression was 17% (RR: 1.17, CI: 1.10 to 1.27, *p* < 0.01) and 77% (RR: 1.58, CI: 1.33 to 1.89, *p* < 0.01) higher compared to individuals without depression. Similarly, the risk of stroke was higher in untreated depression (RR: 1.97, CI: 1.31 to 2.95, *p* < 0.01) compared to treated depression (RR: 1.38, CI: 1.00 to 1.91, *p* = 0.05) with reference to NAFLD individuals without depression. However, the risk of CKD was not increased in both treated and untreated depression (RR: 1.02, CI: 0.84 to 1.26, *p* = 0.82; RR: 1.02, CI: 0.88 to 1.18, *p* = 0.76, respectively). In the analysis of all-cause mortality, both treated (RR: 1.41, CI: 0.98 to 2.04, *p* = 0.07) and untreated depression (RR: 1.68, CI: 1.21 to 2.33, *p* = 0.01) were at increased risk of all-cause mortality. There were no violations of the cox proportional model (Figure 5). Additionally, CVD mortality was significantly increase in untreated depression (SHR: 1.66, CI: 0.94 to 2.93, *p* = 0.08) but not in treated depression (SHR: 0.84, CI: 0.52 to 1.35, *p* = 0.46). Cancer-related mortality was similarly increased in treated and untreated depression (SHR: 1.63 CI: 1.23 to 2.10, *p* < 0.01 and SHR: 1.61, CI: 0.93 to 2.79, *p* = 0.09).

## Discussion

### Main discussion

Depression is common in chronic diseases and can affect treatment compliance, prognosis, and most importantly, a holistic care for patients (30). Evidence from the previous meta-analysis (10) has found a significant association between depression and NAFLD. And the potential mechanism underlying the findings may be explained by the strong ties between hepatic steatosis and insulin resistance, which can interfere with insulin signaling in brain mechanisms (31). Inflammatory markers and pro-inflammatory cytokines, including tumor necrosis factor-alpha and interleukin-6 may also potentiate mood disorders (32). The serotonin pathway can be affected in this group of patients as the expression of monoamine oxidase-A, one of the enzymes catalyzing monoamines like serotonin, has been shown to be increased in NASH (33). Moreover, the compounded presence of concomitant diabetes and obesity commonly associated with NAFLD can intensify the state of inflammation and oxidative stress resulting in a higher risk of depression (34–36).

In the current examination of the NHANES study from 2000 to 2018, we found that up to 30% of patients with NAFLD can be affected by depression with a 12% increase in the risk of depression compared to non-NAFLD individuals in multivariate analysis. Unsurprisingly, individuals who are older, females and Caucasians were at a significantly increased risk of depression in NAFLD. Previous literature has suggested that the increased susceptibility of older adults to depression might be attributed to the associated increase in the prevalence of physical factors such as chronic diseases, organic brain diseases, malignancy, and psychosocial factors (37, 38). The higher risk for depression in females is corroborated by previous literature which highlighted the differences in hormonal changes between males and females (39, 40). In addition, Caucasians displayed a greater risk for depression as reflected in previous studies (41, 42). While some studies have reported that Hispanics and African Americans are more likely to present with depression compared to Caucasians, the difference could be due to disparities in the level of accessibility to mental health services (43). The presence of depression can significantly affect compliance (44) and prognosis in chronic disease (45). In NAFLD, Tomeno et al. found that the presence of depression resulted in a decrease benefits in standard care treatment with no reduction in steatosis markers after 48 weeks compared to NAFLD without depression (46). Also, similar to Sayiner et al., we found that NAFLD individuals with depression increase the rate of all-cause mortality (16). In addition, the cardiovascular burden was significantly higher in depressed individuals where depression increases the risk of CVD and stroke events. There is significant evidence in observational studies between NAFLD and CVD (47, 48), similarly, depression is closely associated with CVD and subsequent complications (49, 50). While the risk cannot be completely mitigated, treated depression was, however, associated with a reduced magnitude of risk of CVD events and all-cause mortality. Treated depression in fact resulted in a non-significant difference in CVD mortality, which is known to be a leading cause of death in NAFLD.

Various guidelines in chronic diseases, including diabetes, obesity, and coronary heart disease have emphasized the importance of periodic screening for depression (51–54). However, clinical practice guidelines in NAFLD have yet to emphasize the importance of psychological wellbeing and screening despite emerging evidence alluding to significant associations between the two diseases (10). Instead, a quick screening of depression can be easily conducted in clinics with widely used diagnostic tools such as the PHQ-9 scale (22, 55). Up to 56 and 90% of NAFLD may suffer from concomitant diabetes and obesity, respectively, which are risk factors in themselves for the development of depression and the risk of depression (56–59). The presence of depression also has profound implications on interventional therapies for NASH. While bariatric procedures have been proposed as a possible treatment for patients with NASH (60), these procedures themselves have also been associated with an increased risk of suicide, postulated to be due to the inability to rely on the coping mechanisms of overeating in some of these patients after bariatric procedures (61). Hence, a multidisciplinary team committed to NAFLD (16), therefore, is essential to allow for the encapsulation of all aspects of care beyond medical needs, and a greater emphasis on psychosocial wellbeing should be emphasized in future guidelines.

### Strength and limitations

The present study provides a comprehensive analysis of the impact of depression on NAFLD. However, there are several limitations. The current analysis is observational in nature and cannot be used to draw causality inferences but rather to show the association between diseases. Nevertheless, it has been described that the association between the two disease entities is complex and likely bi-directional due to the central and peripheral inflammatory response of both diseases (10). The current limitations within the NHANES 2000–2018 dataset have also introduced some limitations. Firstly, diagnosis of hepatic steatosis was limited to FLI and US-FLI as imaging-based diagnosis was unavailable for the NHANES 2000–2018 dataset. Still, the FLI has been widely employed and validated (62) in population-based studies for liver steatosis (36, 63, 64). In addition, the use of antidepressants for a diagnosis of depression in a proportion of the population could result in the presence of a string bias in the evaluation of the results.

## Conclusion

Depression is a major issue in NAFLD patients but has tended to be overlooked and forgotten whose presence can increase the risk of all-cause mortality and cardiovascular disease. While treatment of depression does not result in a complete elimination of risk, there was a reduction in the magnitude of the effect associated with treated depression. Early screening of depression in high-risk individuals should be encouraged to improve the wellbeing of individuals with NAFLD.

## Data availability statement

Publicly available datasets were analyzed in this study. This data can be found here: https://wwwn.cdc.gov/nchs/nhanes/.

## Ethics statement

Ethical review and approval was not required for the study on human participants in accordance with the local legislation and institutional requirements. Written informed consent for participation was not required for this study in accordance with the national legislation and the institutional requirements.

## Author contributions

CN and NC: conceptualization and design. CN, JX, NC, and YC: acquisition of data and analysis and interpretation of data. CN, JX, NC, YC, KC, JQ, WL, DT, RL, CT, AT, XG, BN, NS, DY, NT, DH, MS, MN, MM, and AS: writing – original draft and writing – review and editing. MM: guarantor of the manuscript. All authors approved the final version of the manuscript and agreed to be accountable for the work, ensuring that questions related to the accuracy or integrity of any part of the work are appropriately investigated and resolved.
